# Knockout of Ca_V_1.3 L-type calcium channels in a mouse model of retinitis pigmentosa

**DOI:** 10.1038/s41598-021-94304-3

**Published:** 2021-07-26

**Authors:** Irem Kilicarslan, Lucia Zanetti, Elena Novelli, Christoph Schwarzer, Enrica Strettoi, Alexandra Koschak

**Affiliations:** 1 Institute of Pharmacy, Pharmacology and Toxicology, Center for Chemistry and Biomedicine, University of Innsbruck, Innrain 80-82, 6020 Innsbruck, Austria; 2 CNR Neuroscience Institute, 56124 Pisa, Italy; 3 Department of Pharmacology, Medical University of Innsbruck, 6020 Innsbruck, Austria; 4 Istituto Di Neuroscienze CNR, Area della Ricerca, Via Giuseppe Moruzzi 1, 56100 Pisa, Italy

**Keywords:** Neuroscience, Physiology

## Abstract

Retinitis Pigmentosa is a genetically heterogeneous, degenerative retinal disorder characterized by gradual dysfunction and death of photoreceptors, first rods and later cones, and progressive blindness. Studies suggested that application of L-type calcium channel blockers rescues photoreceptors in paradigms related to Ca^2+^ overflow. To investigate whether Cav1.3 L-type channels have protective effects in the retina, we established a new mouse model by crossing rd10, modeling autosomal-recessive RP, with Cav1.3 deficient mice (rd10/Cav1.3KO). Our immunohistochemical analyses revealed an influence of Cav1.3 channels on the degenerative process of photoreceptors. The absence of Cav1.3 delayed the centre-to-periphery degeneration of rods indicated by a significantly higher number of photoreceptor rows and, consequently, of cones. In accordance with a preserved number of cones we observed a regular row of cone somas in rd10/Cav1.3-KO retinas. Surviving rod photoreceptors maintained synaptic contacts with rod bipolar cells. However, the delay in degeneration was only observed up to postnatal day 45. Although we observed a reduction in the spontaneous oscillatory retinal activity during multielectrode array analyses, measurable functional preservation was lacking in behavioural tests. In conclusion, Cav1.3 channels contribute to photoreceptor degeneration in rd10 retinas but photoreceptor temporary rescue might rather be achieved indirectly through other retinal cell layers.

## Introduction

Retinitis pigmentosa (RP) refers to a group of genetically heterogeneous diseases characterized by a progressive degeneration of photoreceptors and consequent vision loss. Almost 5% of the recessive forms of RP in humans is caused by a nonsense mutation in the gene encoding the beta subunit of the rod photoreceptor cGMP phosphodiesterase 6 (PDE6b)^1^. Pde6rd1 and Pde6rd10 (rd1 and rd10) mice carry two different spontaneous mutations in the rod-PDE6 gene and are established models for human RP^2–4^. Rd1 mice show a fast onset of the disease; starting from post-natal day (P) 8 rods degenerate rapidly and are completely lost at P20^5,6^. Rd10 mice better mimic the human disease because they display a slower onset of rod degeneration, starting at P21, when the retinal synaptogenesis has already ended, and being complete at around P60, when almost no photoreceptors are detectable^4,7^. As in human RP, cone degeneration follows rod cell death in both animal models^8^. PDE6 is an essential player for initiating the phototransduction cascade. After light detection, PDE6 hydrolyses cGMP leading to the closure of cyclic nucleotide-gated channels (CNGC), which triggers photoreceptor hyperpolarization. Therefore, the loss of PDE6 activity induces cGMP accumulation and consequential constitutive calcium (Ca^2+^) influx in the outer segments, both eventually causing cell death^9–11^. CNGCs are the major source of Ca^2+^ influx in the photoreceptor outer segment, while L-type Ca^2+^ channels (LTCCs) are dominant in the cell body and at the synaptic terminal. Cav1.4 is the principal isoform expressed at the active zone of the ribbon synapse, but also Cav1.3 has been detected in photoreceptors^12–17^. Cav1.3 is also expressed in the retinal pigment epithelium (RPE^18^). Different studies tested LTCC blockers in RP animal models with the scope of decreasing Ca^2+^ influx and slowing the degeneration of photoreceptors. Frasson et al. were the first to report a partial delay in the photoreceptor degeneration of rd1 mice using the LTCC blocker D-cis-diltiazem^19^. Sanges and colleagues^20^ found that systemic administration of D-cis-diltiazem decreased the number of apoptotic nuclei and downregulated calpain activity in rd1 mice. Literature regarding the effects of diltiazem is, however, controversial because other studies showed no beneficial action of this drug in rd1 mice^21,22^, in rhodopsin P23H transgenic rats (carrying a dominant opsin mutation^23^), as well as in the rcd1 dog model (carrying a stop codon in the PDE6b gene^24^). The dihydropyridine Ca^2+^ channel blocker nilvadipine reduced photoreceptor degeneration in RCS rats (carrying a mutation in a receptor tyrosine kinase gene^25,26^) and rd1 mice^21^. One attempt to genetically decrease LTCC mediated Ca^2+^ influx in photoreceptors was made by Schön et al. who cross-bred rd1 with Cav1.4- knock out (KO) mice^27^. However, blocking Ca^2+^ currents mediated by Cav1.4 might be counterproductive for restoring vision because Cav1.4-KO mice are functionally blind^28^, and humans with mutations in the *CACNA1F* gene encoding Cav1.4 channels suffer from congenital stationary night blindness type 2 (OMIM 300071, for review^29^). Yet, the study supported a role for LTCC mediated Ca^2+^ influx in the degenerative process of the disease and a short-term preservation of photoreceptors in the rd1 mouse following genetic deletion of the synaptic Cav1.4 channels^27^. To investigate whether targeting another LTCC subtype would have similar protective effects on photoreceptors, we crossbred the rd10 with Cav1.3-KO mice. Cav1.3-KO mice are congenitally deaf due to the absence of L-type Ca^2+^current in the cochlear inner hair cells^30^. Moreover, they exhibit sinoatrial node dysfunctions such as bradycardia and arrhythmia which disappears during physical activity^30^. We chose Cav1.3 for our study because Cav1.3 deficiency does not lead to detectable changes in the basic retinal architecture and does not alter the distribution of Cav1.4 in the retina^31^. Indeed, both the scotopic and photopic ERG of Cav1.3-KO mice show only a slightly reduced b-wave, hence, allowing us to exclude a major visual dysfunction.

In our study, we compared rd10 and rd10/Cav1.3-KO retinas at different time points following the main degeneration stages in the rd10 mouse model^7,32–34^. Our data showed that Cav1.3 contributes to the photoreceptor cell death, however, its elimination is not sufficient to restore retinal function.

## Materials and methods

### Animals

Animals were housed in groups of 2–6 per cage under standard laboratory conditions (12:12 light/dark, lights on at 07:00 h, 22 ± 2 °C, 50–60% humidity) with pelleted food and water available ad libitum. Experimental procedures were designed to minimize animal suffering and the number of used animals and approved by the national ethical committees on animal care and use: the Austrian Federal Ministry for Science and Research and the Italian Ministry of Health (Protocol #14/D-2014, CNR Neuroscience Institute). All methods were performed in accordance with the relevant guidelines and regulations.

### Cav1.3 and rd10 mouse lines

To generate our new double mutant rd10/Cav1.3-KO we cross-bred Pde6b^rd10^ mice (rd10, Jackson Laboratory; genetic background C57BL/6J) and Cav1.3-KO mice (genetic background C57BL/6N^30^) until homozygosity for rd10. For Cav1.3 heterozygosity was kept to generate inbreed controls (rd10/Cav1.3−/− and rd10Cav1.3+/+, referred as rd10/Cav1.3-KO and rd10, respectively). All animals used in the experiments have the same mixed C57BL/6J x C57BL/6N background. Rd10 genotyping was performed with the following primers: forward: GGC CAG TGA GAA CAA GGA AC and reverse: TGA TTC ATC TAG CCC ATC CA (Jackson Laboratory) and the product was digested with *Kasl* restriction enzyme. Cav1.3 genotyping was carried as previously described in^30^. The number of animals used in all the experiments is indicated in Supplementary tables 1 2.

### Tissue preparation and immunohistochemistry

Mice aged P45 and P60 were anesthetized with isoflurane (Vetflurane®k, Virbac) or with intraperitoneal Avertin (3-bromo-ethanol in 1% tert-amyl alcohol; 0.1 ml/5 g body weight) and euthanized by quick cervical dislocation. The following steps were conducted as previously described^35^. In brief, eyes were fixed with 4% paraformaldehyde (PFA) in buffer (either 1X PBS or 0.1M phosphate buffer, pH 7.4) for 1 h. After removal of cornea, lens and vitreous the eye cups were washed four times with buffer and cryoprotected by a 30% sucrose in buffer overnight. Eyecups were orientated along the dorsoventral axis, embedded in OCT Medium (Tissue-Tek O.C.T Compound; Sakura Finetek, Tokyo, Japan) and frozen in liquid nitrogen. Vertical sections (14 µm) were cut on a cryostat (Leica Microsystems, Wetzlar, Germany), mounted on gelatine-coated slides and stored at − 20 °C. For immunofluorescence experiments, sections were washed three times in buffer containing 0.1% Triton X-100 (Sigma-Aldrich, St. Louis, MO, USA), blocked for two hours in buffer with triton containing 1% bovine serum albumin (BSA, Sigma-Aldrich, A7030) and incubated overnight at 4 °C with primary antibodies at concentrations listed in Supplementary table 3. After washing three times with buffer with triton, sections were incubated with the secondary antibodies (Supplementary table 4) for two hours at room temperature. After additional washes, sections were stained with DAPI (1:10,000; Sigma, D-9542) or Hoechst (1:1000) and eventually mounted using Poly/Mount (Polysciences, Inc., Warrington, PA, USA).

Sections were imaged with a Zeiss Imager.Z2 microscope equipped with an Apotome 2 device (Zeiss, Milan, Italy) using an EC Plan-Neofluar 20x/0.50 M27 objective and with a confocal laser scanning microscope (Leica TCS SP5-II; Leica Microsystems, Wetzlar, Germany) using a 40x/1.30 objective. Series of micrographs were taken at 1 and 0.42 µm intervals z-projections with maximum intensities in ImageJ (National Institutes of Health, Bethesda, Maryland, USA). The analysis of GFAP signal were measured using Matlab (version R2018b; The Mathworks Inc., MA, USA. The quantification of cell row numbers and cone cell numbers as well as qualitative analysis of rod bipolar cells were conducted using ImageJ. To better demonstrate fine structures in the outer nuclear layer of rd10 and rd10/Cav1.3-KO retinas, images were post-processed (sharpen filter) in ImageJ (version 1.52a, http://imagej.nih.gov/ij). Images were adjusted for contrast, brightness using ImageJ and finally assembled in Adobe Photoshop CS5.

The number of nuclear cell rows in the outer nuclear cell layer was quantified in the central and far-peripheral regions of dorsal and ventral retinas. Vertical images covered a linear extension of 350–400 µm retinal tissue (frame), which was divided in 7 equally spaced regions. Nuclei were counted along virtual lines intercepting these regions and the average number per frame was then annotated. A minimum of 3 frames of every region from at least two retinal sections was imaged and quantified. Cone cell nuclei with the pedicles were quantified in the frames of vertical sections of 100 µm linear retinal length in the central and peripheral regions of dorsal and ventral retinas. The length of cones was measured in Image J by drawing a line spanning the vertical linear width of the cone cell from the tip to the cone cells terminal in the OPL. Non-adjacent cone cells were randomly picked at different positions to account for variability in cell width. A minimum of 2 frames per retinal location was quantified. Quantification of glial fibrillary acidic protein (GFAP) immunoreactivity was analysed using maximum intensity projections of stacks (5 µm thick) of the retina central area. The quantification was carried with a custom-made Matlab script: the intensity projection images were binarized, and the pixels count above the background threshold was defined as GFAP signal and calculated as pixels per µm. Since the noise staining varied greatly between genotypes the background threshold was newly set for each image in order to reduce nonspecific background staining. The ratio of the GFAP signal in Cav1.3-KO, rd10 and rd10/Cav1.3-KO were calculated by comparison with the GFAP signal in the wild type retina. To quantify the density of rod bipolar cell dendritic arborisation in PKCα-stains, maximum intensity projections of 5 µm image stacks were taken from the central and peripheral retinas. PKCα positive pixels in the outer plexiform layer were traced manually along a 100 µm linear retinal region of each frame. In Image J, image threshold adjustment was applied automatically to the region of interest (ROI) subtracting the nonspecific background. The area was converted to µm^2^ and data from different ROI was averaged for each retina.

### Microelectrode array recordings

Mice were dark-adapted for at least 2 h before the experiment and sacrificed by cervical dislocation after isoflurane anaesthesia. Animals were in the age of P45, P60 and P90 at the time of the experiment. The ventral position on each eye was marked with a permanent marker before excision, and the eyes were put in bath solution (in [mM]: 110 NaCl, 2.5 KCl, 1 CaCl_2_, 1.6 MgCl_2_, 10 D-Glucose, and 22 NaHCO_3_; bubbled with 5% CO_2_/95% O_2_) for dissection. After cornea and lens removal, the retina was isolated and only the dorsal quarter was used for the recordings; the dorsal part was chosen based on cone opsin spectral distribution and visual stimulation spectra. All operations were performed under dim red-light conditions.

Recordings were carried out with perforated 120-electrode micro-electrode arrays (MEA; 120pMEA100/30iR-Ti-pr, Multichannel Systems, Reutlingen, Germany). Experiments were performed as described previously^35^. Briefly, the dorsal central or peripheral retina was placed ganglion cell-side down in the recording chamber. The tissue was continuously perfused with fresh bath solution at 30 °C. Raw data were recorded at 25 kHz with a MEA-system (MEA2100, Multichannel Systems, Reutlingen, Germany). Light stimulation was performed with a computer-controlled digital light processing projector (Lightcrafter E4500MKII, EKB Technologies Ltd, Bat Yam, Israel). Built-in blue and green LED were used that match well the Rhodopsin and M opsin spectra. The light path was integrated with two sets of neutral density (ND) filters (63–390, 63–393, 63–395, Edmund Optics, York, UK) that allowed us to set scotopic (ND8) and eventually photopic (ND4) light stimuli. We presented the same set of visual stimuli at each ND-level during an experiment. Full-field flashes consisted of 1-s negative and positive contrast steps (50% Weber contrast) with 5-s of background grey (grey value 200) in between.

Retinal spikes were extracted from high-passed filtered (500 Hz, 10th-order butterworth filter) traces using Matlab (version R2015a; The Mathworks Inc., MA, USA). Spike sorting was carried out as described^35^, using a custom-made Matlab script. For the oscillation, raw data were low-pass filtered at 50 Hz to obtain the local field potential (LFP). The LFP fundamental frequency was extrapolated from the highest peak of the Fast Fourier transformation of 10 s trace between 2 and 100 Hz.

### Light–dark test

The light/dark transition box was used to compare the visual behaviour. The testing arena (50*50*25 cm) was divided in two compartments: light compartment (400 lx; 2/3 of the box) and a dark compartment (1/3 of the box) communicating with a small opening of 12*12 cm. A camera was placed directly above the apparatus. Only the light compartment was recorded. Each mouse was placed in the lit area, facing the hole to the dark compartment and allowed to freely ambulate in the box for 10 min. We measured the preference of the mouse to stay in the light versus the dark compartment. To be considered in the lit area, the mouse had to completely leave the dark compartment. Experiments were done in accordance with the ARRIVE guidelines. For the analysis a custom-made Matlab (R2018b; The Mathworks Inc., MA, USA) script was used.

### Statistical analysis

Data are presented as mean ± SEM, unless stated otherwise for the indicated number of experiments or cells analysed (n) from the indicated number of animals (N). Data analysis was performed using Clampfit 10.2 (Axon Instruments), Matlab (The Mathworks Inc., MA, USA) and GraphPad Prism 5 (GraphPad Software; Version 9.1.2 (226)). Means per animal were considered normally distributed if the single data points showed a normal distribution. Data were analysed using the Mann–Whitney *U* test, a one-way ANOVA with Bonferroni or Holm-Sidak post hoc tests or a two-way ANOVA with Tukey post hoc test as appropriate and indicated for the individual experiments. Statistical significance was set at *p* < 0.05. Significance levels of *p* < 0.05, < 0.01, or < 0.001 are denoted in graphs by a single, double, or triple asterisk, respectively.

## Results

This study investigated the effect of a Cav1.3 LTCC knockout in the rd10 mouse model of RP. Previous studies suggested that LTCC blockage or genetic ablation could rescue photoreceptor function in paradigms of RP caused by Ca^2+^ overflow^27,36^. To investigate the impact of Cav1.3 LTCCs on the retinal degeneration caused by the rd10 mutation, we established a new mouse model by crossbreeding rd10 mice with Cav1.3 deficient mice (Cav1.3-KO) to obtain mutant rd10/Cav1.3-KO mice.

### Cav1.3 deficiency delays photoreceptor degeneration in rd10 mice

**Figure 1:**
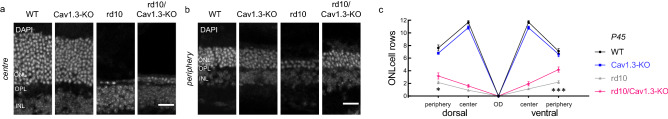
Outer nuclear layer measurements in the central and peripheral regions at P45. Nuclear staining with DAPI (grey) in the outer nuclear layer (ONL) of central **(a)** and peripheral **(b)** regions in wild type, Cav1.3-KO, rd10 and rd10/Cav1.3-KO retinas at P45. (**c**) For spider plot analysis, the numbers of ONL rows of Cav1.3-KO, rd10 and rd10/Cav1.3-KO were compared to wild type in the center and periphery of dorsal or ventral retinas. Dorsal: centre: wild type (WT): 11.7 ± 0.3; Cav1.3-KO: 10.9 ± 0.3; rd10: 0.9 ± 0.1; rd10/Cav1.3-KO: 1.6 ± 0.2; periphery: wild type: 7.6 ± 0.5; Cav1.3-KO: 6.8 ± 0.2; rd10: 2.1 ± 0.2; rd10/Cav1.3-KO: 3.2 ± 0.5. Ventral: centre: wild type (WT): 11.7 ± 0.2; Cav1.3-KO: 10.8 ± 0.3; rd10: 1.1 ± 0.1; rd10/Cav1.3-KO: 2 ± 0.3; periphery: wild type: 7.6 ± 2.1; Cav1.3-KO: 7 ± 2; rd10: 1.3 ± 0.4; rd10/Cav1.3-KO: 2.2 ± 0.7. N = 4 for all strains. Statistics: *, *p* < 0.01, ***p < 0.001, Two-way ANOVA with Tukey multiple comparison test. Only the comparison between rd10 and rd10/Cav1.3-KO is indicated in the figure. There was no significant difference between wild type and Cav1.3-KO, and *** significance between either wild type or Cav1.3-KO and the two rd10 models. Data are presented as means ± SEM. *Scale bars: 20 µm.*

First, we investigated changes in the thickness of the outer nuclear layer (ONL) at P45, the peak of cone cell death that follows the peak degeneration of rods. Because the rd10 mutation primarily affects rod photoreceptors (making up 97% of all photoreceptors in the mouse retina^37^, and the fact that the degeneration wave follows a centre-to-periphery gradient^7^, we counted the cell rows in the central and peripheral ONL of rd10 and rd10/Cav1.3-KO retinas and compared them with Cav1.3-KO and wild type (Fig. 1). In dorsal and ventral regions, rd10 and rd10/Cav1.3-KO mice showed a significant reduction of cell nuclei in the ONL compared to wild type and Cav1.3-KO controls (Fig. 1c). In the central retina, the two rd10 models showed no statistical difference in the number of photoreceptor rows while we observed a slight but significant reduction in Cav1.3-KO mice compared to wild type (Fig. 1b). In the periphery, instead, where WT and Cav1.3-KO have comparable ONL width, we detected significantly more cell rows in the rd10/Cav1.3-KO retina than in rd10 (Fig. 1c). We therefore explain the difference in ONL thickness in the centre and periphery as the consequence of a delayed degeneration of peripheral photoreceptors (mostly rods) in the absence of Cav1.3 LTCCs in the rd10 retina.

### Cone cell degeneration was delayed in rd10/Cav1.3-KO mice

**Figure 2:**
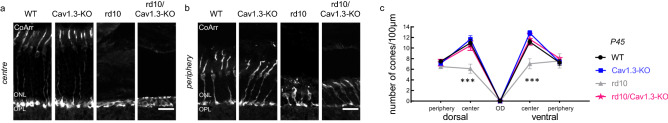
Number of cones in the central and peripheral regions at P45. Cones stained against cone arrestin in the central **(a)** and peripheral **(b)** retina of wild type, Cav1.3-KO, rd10 and rd10/Cav1.3-KO mice. (**c**) For spider plot analysis, the number of cones in 100 µm linear retinal length was counted in the center and periphery of dorsal or ventral retinas. Dorsal: centre: wild type (WT): 11 ± 0.6; Cav1.3-KO: 11.7 ± 0.7; rd10: 6.1 ± 0.9; rd10/Cav1.3-KO: 10.5 ± 0.9; periphery: wild type: 7.5 ± 0.3; Cav1.3-KO: 7.1 ± 0.2; rd10: 6.5 ± 0.4; rd10/Cav1.3-KO: 7.2 ± 0.6. Ventral: centre: wild type (WT): 11.2 ± 0.5; Cav1.3-KO: 12.8 ± 0.5; rd10: 7 ± 0.9; rd10/Cav1.3-KO: 11.7 ± 1.1; periphery: wild type: 7.3 ± 0.6; Cav1.3-KO: 7.3 ± 0.3; rd10: 7.6 ± 1.3; rd10/Cav1.3-KO: 8 ± 0.5. N = 3 for all strains. Statistics: ***p < 0.001. Two-way ANOVA with Tukey multiple comparison test. Only the comparison between rd10 and rd10/Cav1.3-KO is indicated in the figure. There was no significant difference between wild type and Cav1.3-KO and rd10/Cav1.3-KO. Data are presented as means ± SEM. Scale bars: 20 µm.

At P45, when only a single layer of photoreceptors is left in rd10 mice, we also quantified cones and compared their morphology in all four mouse strains (Fig. 2). Similar to previous studies^38^, we observed a significant reduction of cones in the dorsal and ventral central retina of rd10 mice (Fig. 2c). Rd10/Cav1.3-KO retinas, instead, showed a comparable number of cones in wild type and Cav1.3-KO controls (Fig. 2c). In the periphery, the number of cones was unchanged in all four mouse strains (Fig. 2c).

**Figure 3:**
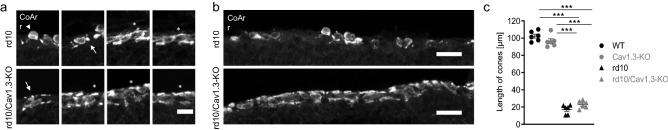
Cone morphology in the central retina at P45. (**a)** Cone outer segments were observed in variable forms in both rd10 and rd10/Cav1.3-KO strains as shorter (asterisks), circular (arrows) and absent (arrowheads). **(b)** Cone soma organization in rd10 and rd10/Cav1.3-KO retinas in the central region. **(c)** The length of 20 cones within different segments of 300 µm linear length of central retinas was measured [in µm]: wild type (WT): 102.3 ± 2.3; Cav1.3-KO: 96.4 ± 2.8; rd10: 17.2 ± 2.1; rd10/Cav1.3-KO: 22.9 ± 1.8. N = 6 for all strains. Statistics: *** p < 0.001. One-way ANOVA with Holm-Sidak’s multiple comparison test. Data are presented as means ± SEM. Scale bars: 10 µm (**a**), 20 µm (**b**).

While Cav1.3-KO cones preserved their typical morphology with a long outer segment (OS), cell body, axon and pedicle structures, rd10 and rd10/Cav1.3-KO cones were severely affected (Fig. 3). Rd10 cones mostly lacked axons and their OSs had a circular shape or were absent with smaller pedicles in the central retina^32^ (Fig. 2a,3a). In the central area of rd10/Cav1.3-KO mice, cone axons were still detectable but abnormal OSs were also observed: they were mainly short or circular, or (rarely) absent (Figs. 2a,3a). In the peripheral regions of both rd10 and rd10/Cav1.3-KO retinas, axons and OSs appeared longer (Fig. 2c). In accordance with a preserved number of cones in rd10/Cav1.3-KO retinas also the organization of their somas in the ONL was different from the rd10. While in rd10 the cone arrestin pattern appeared scattered or aggregated in clusters, the rd10/Cav1.3-KO displayed a regular row of cone somas adjacent to the OPL (Fig. 3b). We did not a find a significant change in the overall length of the cones due to the absence of Cav1.3 (Fig. 3c). Still, our observations collectively corroborate an influence of Cav1.3 LTCCs on the degeneration of photoreceptors, because the absence of Cav1.3 delayed the centre-to-periphery degeneration of rods, as indicated by the higher number of photoreceptor rows (Fig. 1 b, d) and consequently of cones.

### Glial activation was not reduced in rd10/Cav1.3-KO

**Figure 4:**
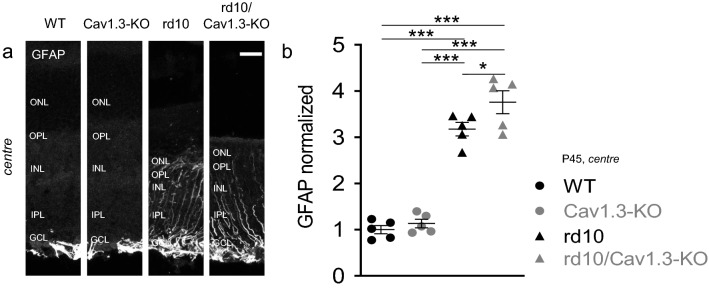
Glial fibrillary acidic protein signal in the central region at P45. **(a)** Immunoreactivity against glial fibrillary acidic protein illustrates Müller glia cell activation in wild type, Cav1.3-KO, rd10 and rd10/Cav1.3-KO retinas in the central region at P45. **(b)** The GFAP signal in the central region of Cav1.3-KO, rd10 and rd10/Cav1.3-KO retinas was normalized to wild type: Cav1.3-KO: 1.1 ± 0.1; rd10: 3.2 ± 0.1; rd10/Cav1.3-KO: 3.8 ± 0.2. N = 5 for all strains. Statistics: ***p < 0.001. One-way ANOVA with Holm-Sidak’s multiple comparison test. Data are presented as means ± SEM. Scale bars: 20 µm.

Similar to other retinal pathologies, glial reaction follows photoreceptor cell death^39^. Glial fibrillary acidic protein (GFAP) is upregulated upon glial activation in the mouse retina^40^ and constantly upregulated in rd10 mice in Müller cells^7^. Because GFAP accumulation is found in Müller cells of peripheral wild type retinas (and also in Cav1.3-KO, data not shown) unrelated to glial hyperreactivity^41^, we focused on the central retina of P45 mice. As shown in Fig. 4a, the anti-GFAP antibody labelled Müller cells throughout all retinal layers in rd10 and rd10/Cav1.3-KO retinas. Compared to wild type and Cav1.3-KO controls we found significantly higher GFAP immunoreactivity in both rd10 and rd10/Cav1.3-KO retinas. Hence, knocking out Cav1.3 in rd10 retinas increased photoreceptor survival but did not reduce GFAP accumulation (Fig. 4b).

### Rod bipolar cell dendrites in rd10/Cav1.3-KO mice

**Figure 5:**
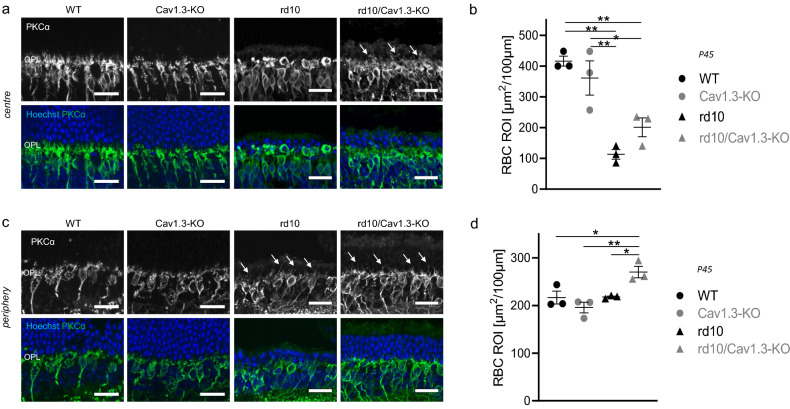
Rod bipolar cell dendritic arbours. Co-immunostaining with PKCα (green) and Hoechst (blue) nuclear staining (blue) shows rod bipolar cell dendritic arbours and the cell nuclei respectively in the central **(a)** and peripheral **(c)** regions of wild type, Cav1.3-KO, rd10 and rd10/Cav1.3-KO retinas at P45. Rod bipolar cells lack dendritic trees in the central region of rd10 retinas. Few dendritic sprouts into the ONL (arrows) were identified in the central region of rd10/Cav1.3-KO retinas and in the periphery of both rd10 strains. **(b)** and **(d)** shows the quantification of dendritic arborizations in central **(b)** and peripheral **(d)** retinas [in µm^2^]: centre: wild type (WT): 416 ± 16; Cav1.3-KO: 361 ± 56; rd10: 112.9 ± 15.7; rd10/Cav1.3-KO: 201 ± 30.7; periphery: wild type: 216.7 ± 13.6; Cav1.3-KO: 195.7 ± 11.3; rd10: 217.8 ± 2; rd10/Cav1.3-KO: 270.2 ± 12.1. N = 3 for all strains. Statistics: ***p < 0.001 in centre; **p = 0.007 in periphery, One-way ANOVA with Holm-Sidak’s multiple comparison test. Data are presented as means ± SEM. Scale bars: 20 µm.

Dendritic regression of rod bipolar cells (RBCs) is another morphological feature of rd10 mice^7^ that closely follows rod demise^42^. Therefore, the morphology of rod bipolar cell dendritic arborisations served as an indicator of the preservation of rods. In the central area of rd10 retinas at P45, RBCs are almost devoid of dendritic arborisation (Fig. 5a,b^7,32,33^) while some dendrites are visible in the periphery (Fig. 5b,d^7^), in good agreement with the number of cell rows in the ONL. In contrast, in rd10/Cav1.3-KO retinas, dendrites of rod bipolar cells are clearly discernible in the central retina (Fig. 5a,b) as well as in the periphery (Fig. 5c,d). These observations suggest that surviving rd10/Ca_V_1.3-KO rod photoreceptors maintain synaptic contacts with RBCs.

### Temporal window of rd10 degeneration delay

**Figure 6:**
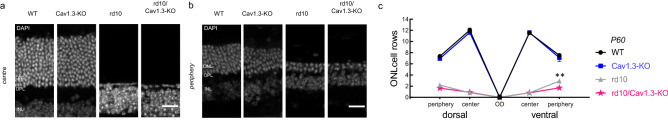
Outer nuclear layer nuclear staining in the central and peripheral regions at P60. Nuclear staining with DAPI in the outer nuclear layer (ONL) of central **(a)** and peripheral **(b)** regions in wild type, Cav1.3-KO, rd10 and rd10/Cav1.3-KO retinas at P60. **(c)** For spider plot analysis at least 5 mice of each genotype were counted. Outer nuclear layer cell row numbers at P60 in the centre and periphery of dorsal and ventral retinas are shown. Dorsal: centre: wild type (WT): 12 ± 0.4; Cav1.3-KO: 11.7 ± 0.2; rd10: 0.8 ± 0.1; rd10/Cav1.3-KO: 0.9 ± 0.1; periphery: wild type: 7.4 ± 0.2; Cav1.3-KO: 7 ± 0.3; rd10: 2.2 ± 0.1; rd10/Cav1.3-KO: 1.7 ± 0.2. Ventral: centre: wild type (WT): 11.6 ± 0.2; Cav1.3-KO: 11.6 ± 0.3; rd10: 0.8 ± 0.04; rd10/Cav1.3-KO: 0.8 ± 0.1; periphery: wild type: 7.5 ± 0.2; Cav1.3-KO: 7.1 ± 0.7; rd10: 2.9 ± 0.2; rd10/Cav1.3-KO: 1.7 ± 0.3. Statistics: ***p < 0.001, Two-way ANOVA with Tukey’s multiple comparison test. Only the comparison between rd10 and rd10/Cav1.3-KO is indicated in the figure. There was no significant difference between wild type and Cav1.3-KO, and *** significance between either wild type or Cav1.3-KO and the two rd10 models. Data are presented as means ± SEM. *Scale bars: 20 µm.*

**Figure 7:**
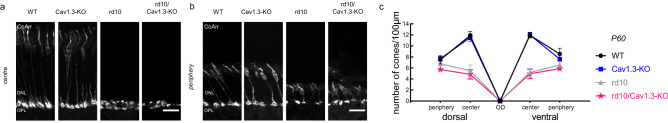
Cone cell numbers and cell morphology at P60 in the central and peripheral regions. Cone arrestin immunoreactivity shows cone cells in the central **(a)** and peripheral regions **(b)** of wild type, Cav1.3-KO, rd10 and rd10/Cav1.3-KO retinas at P45. (**c**) For spider plot analysis at least 5 mice of each genotype were counted. The number of cones in 100 µm linear retinal length was counted in the center and periphery of dorsal or ventral retinas. Dorsal: centre: wild type (WT): 11.9 ± 0.7; Cav1.3-KO: 11.5 ± 0.7; rd10: 5.5 ± 1; rd10/Cav1.3-KO: 4.9 ± 0.8; periphery: wild type: 7.5 ± 0.7; Cav1.3-KO: 7.7 ± 0.4; rd10: 6.8 ± 0.3; rd10/Cav1.3-KO: 5.8 ± 0.4. Ventral: centre: wild type (WT): 11.9 ± 0.5; Cav1.3-KO: 12 ± 0.5; rd10: 5.2 ± 0.7; rd10/Cav1.3-KO: 5 ± 0.8; periphery: wild type: 8.5 ± 1; Cav1.3-KO: 7.6 ± 0.3; rd10: 6.5 ± 0.4; rd10/Cav1.3-KO: 5.9 ± 0.5. There was no significant difference between wild type and Cav1.3-KO and rd10 and rd10/Cav1.3-KO. *** significant difference between either wild type or Cav1.3-KO and the two rd10 models was found (not indicated for a better overview in the figure). Statistics: p = 0.06 in dorsal periphery; p = 0.05 in ventral periphery; ***p < 0.001 in dorsal and ventral centre, Two-way ANOVA with Tukey’s multiple comparison test. Data are presented as means ± SEM. Scale bars: 20 µm.

To investigate the time span of photoreceptor degeneration delay in rd10/Cav1.3-KO retinas, we examined retinas of animals aged P60, as this was described as the time point when the scotopic ERG is extinguished, and only scattered cone cells persist in the ONL^33^. We did not observe, however, significant differences in the central and peripheral ONL of rd10 and rd10/Cav1.3-KO mice (Fig. 6a,b), except for the ventral periphery (Fig. 6c). Also, the number of cones in rd10/Cav1.3-KO retinas was comparable to rd10 (Fig. 7c). P60 cones were lacking their OS and identifiable fibres and pedicles in both rd10 and rd10/Cav1.3-KO in the central area (Fig. 7a); also, the peripheral retinal cone morphology was comparable (Fig. 7b). These results indicate that the delay in photoreceptor degeneration in rd10/Cav1.3KO mice has a limited temporal window.

### Cav1.3 deficiency doesn’t preserve retinal function in rd10 mice

**Figure 8:**
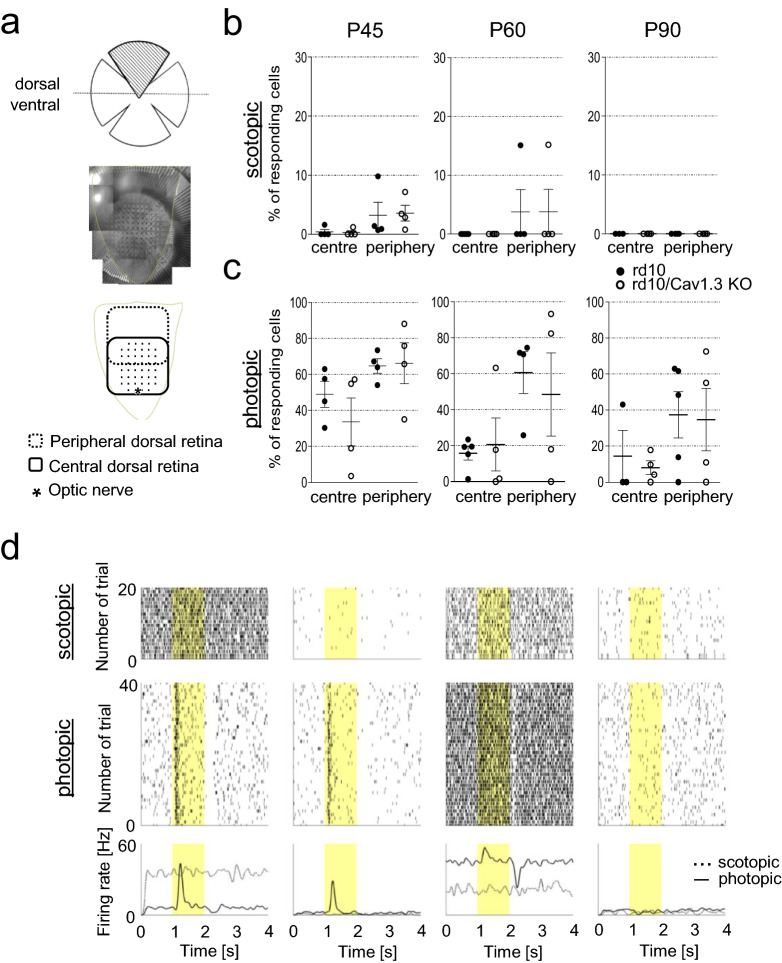
The number of responding ganglion cells is comparable in rd10 and rd10/Cav1.3. **(a)** Schematic representation of the experimental paradigm. Only the dorsal quadrant of the retina was used for the MEA recording. The optic nerve or the retina´s dorsal edge was placed close to the outermost MEA electrodes, defining central and dorsal retina respectively. **(b)** Number of ganglion cells responding in scotopic light in the centre and periphery at P45, P60 and P90. 100% was set to all ganglion cells successfully spike-sorted. P45: rd10: centre = 0.40 ± 0.40, periphery = 3.20 ± 2.20, N = 4; rd10/Cav1.3-KO: centre = 0.29 ± 0.29, periphery = 3.56 ± 1.32, N = 4; P60: rd10: centre = 0, periphery = 3.77 ± 3.77, N = 4; rd10/Cav1.3-KO: centre: = 0; periphery = 3.80 ± 3.80, N = 4; P90: rd10: none responding, N = 4; rd10/Cav1.3-KO, none responding, N = 4. **(c)** Number of ganglion cells responding in photopic light in the centre and periphery at P45, P60 and P90. 100% of the ganglion cells was set of all the spiking ganglion cells successfully recorded. Mean ± SEM. P45: rd10, centre = 48.88 ± 7.26, periphery = 64.62 ± 4.05, N = 4; rd10/Cav1.3-KO: centre = 43.56 ± 12.39, periphery = 76.15 ± 6.47, N = 4; P60: rd10, centre = 14.75 ± 4.78, periphery = 60.60 ± 11.66, N = 4; rd10/Cav1.3-KO: centre = 20.59 ± 14.73, periphery = 48.35 ± 23.15, N = 4; P90: rd10: centre = 14.33 ± 14.33, periphery = 37.29 ± 12.87, N = 3–5; rd10/Cav1.3-KO: centre = 7.84 ± 3.84, periphery = 34.57 ± 17.35, N = 4. For the number of responding cells, see Supplementary table 3. Data are presented as mean ± SEM. No statistically significant differences were observed (Mann Whitney U-test). **(d)** Example of ganglion cells responses to the full field flash stimulus of rd10 retinas at P90. Raster plots of four different ganglion cells showing spiking activity in response to scotopic (top) and photopic (middle) light stimulation (yellow shadowing). Each dot represents an action potential. At the bottom the average convolved spike rates of the scotopic and photopic responses are indicated.

The slower photoreceptor degeneration emerging from our immunohistochemical analysis prompted us to test whether some light-initiated retinal activity was preserved as well. Thus, we evaluated the number of ganglion cells responding to rod-driven (scotopic) or cone-driven (photopic) light stimulation in the periphery and the centre of rd10 and rd10/Cav1.3 KO retinas (Fig. 8), using MEA recordings. In the central retina scotopic responses were absent in both strains at all ages investigated (P45, P60, P90), while in the periphery light responses were only undetectable at P90 (Fig. 8b). On the contrary, we observed photopic responses in the central and peripheral retina in both genotypes at all ages (Fig. 8c,d). The comparison between the number of responding ganglion cells in the various genotypes did not show statistical significance in any condition (centre or periphery versus age, legend of Fig. 8 and Supplementary table 3), indicating a lack of measurable functional preservation.

One of the main features of the degenerating retina is a spontaneous activity that can be observed as rhythmic spiking, and in general, as oscillatory activity of ganglion cells (Supplementary Fig. 1a, for review see^43^). In our MEA experiments we observed that in the periphery rd10/Cav1.3 KO retinas showed a reduced spontaneous activity compared to rd10 (Supplementary Fig. 1b). In the central region we observed a significant reduction of the oscillatory frequency only at P60 (Supplementary Fig. 1b). However, the oscillatory activity did not correlate with the photoreceptor rescue—e.g., at P60 no difference in the number of cones has been observed (Fig. 7).

Yet, a reduction in the spontaneous oscillatory frequency of a degenerating retina might still improve the delivery of the visual information to higher visual centres in the brain^44^. Therefore, we investigated and compared the visual performance of Cav1.3 KO, rd10 and rd10/Cav1.3 KO mice *in-vivo* subjecting them to a vision based behavioural test. Using a light/dark transition box, all Cav1.3-KO mice spent more than 70% of the time in the dark compartment, implying normal vision (Supplementary Table 5). At P27 no statistical difference was observed between the three genotypes. In accordance with the disease development the time spent in the dark dropped to approximately 50% in the rd10 at P45 (Supplementary table 5). The same loss of light avoidance behaviour was observed in rd10/Cav1.3-KO mice; neither rd10 strains showed a clear preference for one of the light conditions at P45. This behavioural experiment indicates that knocking-out Cav1.3 LTCCs is not sufficient to significantly improve the visual performance of rd10 mice.

## Discussion

Delaying rod degeneration in RP is considered a valid therapy as cone survival prolonged consequentially and this improves the life quality of patients per se^45^. The idea of reducing Ca^2+^ influx to slow down the apoptotic machinery has been captivating since the first effective studies using Ca^2+^ channel blockers two decades ago^19,23^. Moreover, two genetic approaches highlighted the potential of reducing Ca^2+^ influx^27,46^. The absence of LTCCs in β2-KO mice resulted in a short-term delay in degeneration in rd1 mice^46^. Similarly, some photoreceptor rescue was achieved when rd1 mice were crossbred with mice lacking Cav1.4 α1 subunits. However, both types of genetically modified mice are functionally blind because synaptic transmission from photoreceptors to bipolar cells is disrupted^47–51^.

Our newly generated rd10/Cav1.3-KO mutant mouse model benefits from the fact that that the channel knock-out per se neither affects retinal architecture or function^31,52^. Indeed, in the absence of Cav1.3 channels some photoreceptors were preserved in rd10 mice. Consequently, at P45 the dendritic arbours of RBCs were still detectable in the central retina of the double mutant, corroborating the observed delay in photoreceptor degeneration. Nevertheless, the effect was transient, because at P60 photoreceptor degeneration in rd10/Cav1.3-KO was comparable to rd10. Similar findings have been reported in studies using Ca^2+^ channel blockers^20,21,36^ or genetic ablation of photoreceptor L-type VGCCs^27,46^. Our data show that the observed effect is an alleviation of the degeneration rather than a true rescue of photoreceptors from death. We can conclude that Cav1.3, as one of the known contributors of Ca^2+^ entry in photoreceptors^12–17^, is a contributor to photoreceptor cell death. However, its absence is not sufficient to fully rescue photoreceptors. In particular, toxic cGMP accumulation by might lead to cell death in rd10/Cav1.3-KO retinas, even in the absence of Cav1.3^53^.

While a better survival of photoreceptors should be reflected in improved vision, we did not observe any difference in the light responses of the two rd10 strains. Two considerations are important to make: (1) both genotypes showed a high internal variability (as also previously shown for rd mice^22,46,54–57^) and (2) we recorded clear ganglion cell responses upon light stimulation at P90 (Fig. 8d). To our knowledge visual function in rd10 mice older than P60 was detected only one other study. In fact, Ivanova et al. used a virtual optokinetic system and detected “low, but measurable vision as late as P160”^58^. Our finding highlights the high sensitivity of MEA in detecting light-driven responses, at the single cell population level, in mice considered virtually blind when recorded with mass methods or tested behaviourally, and indicate that persistency of functional photoreceptors and retinal circuits in rd10 mutants might be longer than estimated.

The lack of visual improvement despite the delayed degeneration of photoreceptors in rd10/Cav1.3-KO retinas might be due to other factors beside the reduction of Ca^2+^ influx in the photoreceptors. We have to consider that Cav1.3 is not the predominant LTCC in the photoreceptors, whereas its expression in the RPE is well documented^18,59–62^. Therefore, we cannot exclude that an increase in photoreceptors survival was due to a change in the activation or expression of trophic factors from the RPE. In RPE cells Cav1.3 is a regulator of phagocytic activity^61^. In addition, Cav1.3 co-immunoprecipitates with the fibroblast growth factor receptor 2, suggesting a tight functional interaction between fibroblast growth factor 2 (FGF2) and the Ca^2+^ channel in these cells^59^. In RCS rats and rd1 mice, administration of nilvadipine has been reported to up-regulate expression levels of FGF2 encoding genes and provided preservation of photoreceptor cells^21,26^. Moreover, application of nilvadipine to retinal pigment cells has been shown to decrease the secretion of vascular endothelial growth factor (VEGF)^63,64^. Of note, anti-VEGF agents are currently in use in clinical studies and showed efficacy in patients with RP associated cystoid macular oedema^65–67^. Along this line, previous studies showing beneficial effects of dihydropyridines in different animal models of RP^21,25,26^ might have interfered with the release of neuronal growth factors. We achieved similar beneficial results by specifically “blocking” the most prominent LTCC in the RPE, rather than delivering pan-LTCC blockers (acting mainly in the peripheral nervous system^68,69^). Although deeper investigations would have exceeded the scope of this study, we emphasize to explore the importance of the RPE in rd10/Cav1.3-KO in the future.

In conclusion, our study shows that Cav1.3 channels contribute to the photoreceptor degeneration in rd10 retinas. Cav1.3 deficiency provides a short-term photoreceptor cell preservation in an RP mouse model but is unable to protect sustained neuronal function. It is possible that photoreceptor temporary rescue is achieved indirectly through the RPE, confirming the neuroprotective role of this important cell layer.

## Supplementary Information

Supplementary Information.

## Data Availability

The datasets generated during and/or analysed during the current study are available from the corresponding authors on reasonable request.
