# Supplementary material for: Knockout of CaV1.3 L-type calcium channels in a mouse model of retinitis pigmentosa
Source: Sci Rep. 2021 Jul 26;11:15146. doi: 10.1038/s41598-021-94304-3 (PMC8313562; doi:10.1038/s41598-021-94304-3)
Supplement: Supplementary file 1 — Supplementary Information. [file 41598_2021_94304_MOESM1_ESM.pdf]

Supplemental material

Supplementary tables

Supplementary table 1: Quantitative and qualitative morphological analyses in wild type, Cav1.3-KO, rd10 and rd10/Cav1.3-KO retinas**.** The number of retinas (N) examined and analysed frames (n) of retinal regions is indicated for the central (centre) and peripheral retina (periphery) at the age of P45 and P60.

**P45**			
**Outer nuclear layer**			
Wild type			
Centre		Periphery	
Retinas (N)	Analysed frames (n)	Retinas (N)	Analysed frames (n)
6	59	6	52
Ca$_V$1.3-KO			
Centre		Periphery	
Retinas (N)	Analysed frames (n)	Retinas (N)	Analysed frames (n)
6	43	6	36
rd10			
Centre		Periphery	
Retinas (N)	Analysed frames (n)	Retinas (N)	Analysed frames (n)
6	28	6	29
rd10/Ca$_V$1.3-KO			
Centre		Periphery	
Retinas (N)	Analysed frames (n)	Retinas (N)	Analysed frames (n)
6	30	6	39
**Cone photoreceptors**			
Wild type			
Centre		Periphery	
Retinas (N)	Analysed frames (n)	Retinas (N)	Analysed frames (n)
6	35	6	33
Ca$_V$1.3-KO			
Centre		Periphery	
Retinas (N)	Analysed frames (n)	Retinas (N)	Analysed frames (n)
6	31	6	30
rd10			

Centre		Periphery	
Retinas (N)	Analysed frames (n)	Retinas (N)	Analysed frames (n)
6	31	6	31
rd10/Ca$_V$1.3-KO			
Centre		Periphery	
Retinas (N)	Analysed frames (n)	Retinas (N)	Analysed frames (n)
6	26	6	36
**Glial fibrillary acidic protein**			
Wild type			
Centre			
Retinas (N)		Analysed frames (n)	
5		5	
Ca$_V$1.3-KO			
Centre			
Retinas (N)		Analysed frames (n)	
5		5	
rd10			
Centre			
Retinas (N)		Analysed frames (n)	
5		5	
rd10/Ca$_V$1.3-KO			
Centre			
Retinas (N)		Analysed frames (n)	
5		5	
**Rod bipolar cells**			
Wild type			
Centre		Periphery	
Retinas (N)	Analysed frames (n)	Retinas (N)	Analysed frames (n)
3	4	3	4
Ca$_V$1.3-KO			
Centre		Periphery	
Retinas (N)	Analysed frames (n)	Retinas (N)	Analysed frames (n)
3	5	3	5
rd10			
Centre		Periphery	
Retinas (N)	Analysed frames (n)	Retinas (N)	Analysed frames (n)
3	10	3	11
rd10/Ca$_V$1.3-KO			
Centre		Periphery	
Retinas (N)	Analysed frames (n)	Retinas (N)	Analysed frames (n)

3	21	3	26
**P60**			
**Outer nuclear layer**			
Wild type			
Centre		Periphery	
Retinas (N)	Analysed frames (n)	Retinas (N)	Analysed frames (n)
6	40	6	32
Cav1.3-KO			
Centre		Periphery	
Retinas (N)	Analysed frames (n)	Retinas (N)	Analysed frames (n)
6	26	6	27
rd10			
Centre		Periphery	
Retinas (N)	Analysed frames (n)	Retinas (N)	Analysed frames (n)
6	33	6	29
rd10/Cav1.3-KO			
Centre		Periphery	
Retinas (N)	Analysed frames (n)	Retinas (N)	Analysed frames (n)
6	24	6	21
**Cone photoreceptors**			
Wild type			
Centre		Periphery	
Retinas (N)	Analysed frames (n)	Retinas (N)	Analysed frames (n)
6	29	6	23
Cav1.3-KO			
Centre		Periphery	
Retinas (N)	Analysed frames (n)	Retinas (N)	Analysed frames (n)
6	26	6	28
rd10			
Centre		Periphery	
Retinas (N)	Analysed frames (n)	Retinas (N)	Analysed frames (n)
6	25	6	23
rd10/Cav1.3-KO			
Centre		Periphery	
Retinas (N)	Analysed frames (n)	Retinas (N)	Analysed frames (n)
6	24	6	22

Supplementary table 2. Ganglion cells rd10 and rd10/Cav1.3 KO mice responding to scotopic and photopic light stimulation in MEA recordings. Number of retinas tested, analysed ganglion cells and responding ganglion cells is indicated for the central (centre) and peripheral retina (periphery).

**P45**									
rd10									
Centre					Periphery				
Retinas (N)	Analysed GC (n)	Responding GC (n)			Retina s (N)	Analysed GC (n)	Responding GC (n)		
4	258	scotopic	1		4	392	scotopic	9	
		photopic	128				photopic	248	
Rd10/Cav1.3 KO									
Centre					Periphery				
Retinas (N)	Analysed GC (n)	Responding GC (n)			Retina s (N)	Analysed GC (n)	Responding GC (n)		
4	341	scotopic	1		4	325	scotopic	9	
		photopic	97				photopic	193	

**P60**									
rd10									
Centre					Periphery				
Retinas (N)	Analysed GC (n)	Responding GC (n)			Retina s (N)	Analysed GC (n)	Responding GC (n)		
5	346	scotopic	0		5	355	scotopic	13	
		photopic	51				photopic	241	
rd10/Cav1.3 KO									
Centre					Periphery				
Retinas (N)	Analysed GC (n)	Responding GC (n)			Retina s (N)	Analysed GC (n)	Responding GC (n)		
4	235	scotopic	0		4	262	scotopic	9	
		photopic	58				photopic	111	

**P90**									
rd10									
Centre					Periphery				
Retinas (N)	Analysed GC (n)	Responding GC (n)			Retinas (N)	Analysed GC (n)	Responding GC (n)		
3	198	scotopic	0		5	261	scotopic	0	
		photopic	43				photopic	123	
Rd10/Cav1.3 KO									
Centre					Periphery				
Retinas (N)	Analysed GC (n)	Responding GC (n)			Retinas (N)	Analysed GC (n)	Responding GC (n)		
4	182	scotopic	0		4	240	scotopic	1	
		photopic	17				photopic	90	

Supplementary table 3: List of Primary Antibodies

**Protein**	**Host Species**	**Working Dilution**	**Source, Catalog Order No.**
Cone Arrestin	Rabbit	1:1000	Sigma Aldrich, AB15282
GFAP	Rabbit	1:1000	Abcam, ab7260
GFAP	Rabbit	1:1000	Sigma-Aldrich, G9269
PKCα	Rabbit	1:800	Sigma-Aldrich, P4334

Supplementary table 4: List of Secondary Antibodies

**Antibody**	**Working Dilution**	**Source, Catalog Order No.**
Rhodamine Red X donkey-anti-rabbit IgG (H+L)	1:400	Jackson Immuno Research 715-295-152
Alexa Fluor® 488 donkey-anti-rabbit IgG (H+L)	1:400	Invitrogen, A-21206
Alexa Fluor® 568 goat-anti-rabbit IgG (H+L)	1:400	Invitrogen, A-11011

Supplementary table 5: Light/dark test at P27 and P45.

The percentage of time spent in the light compartment of Cav1.3-KO, rd10 and rd10/Cav1.3-KO is shown for P27 and P45. While no statistically significant difference between the two tested time-point was observed for Cav1.3-KO, both rd10 and rd10/Cav1.3-KO mice spent significantly more time in the light compartment at P45 compared to P27. Unpaired two-tail t-test between the same genotype (*, $p<0.05$, **, $p<0.01$); two-way ANOVA with Bonferroni´s multiple comparison test for statistical analysis between genotypes at different ages. Note that no statistical difference was observed between the three genotypes at P27, while at P45, rd10 and Cav1.3-KO spent significantly more time in the light compartment compared to Cav1.3-KO ($^{\#}$, $p<0.05$, $^{\#\#}$, $p<0.01$). Time is shown as mean ± SD.

	Time spent in the light compartment [%]		
	P27	P45	**N**
Cav1.3 KO	22.3 ± 13.0	29.4 ± 10.0	**12**
rd10	31.6 ± 11.2	45.8 ± 11.1** $^{\#\#}$	**14**
rd10/Cav1.3 KO	29.5 ± 13.8	42.8 ± 14.8* $^{\#}$	**12**

Supplementary figure

Supplementary figure 1: Spontaneous rhythmic electrical activity in rd10 and rd10/Cav1.3 KO retinas.

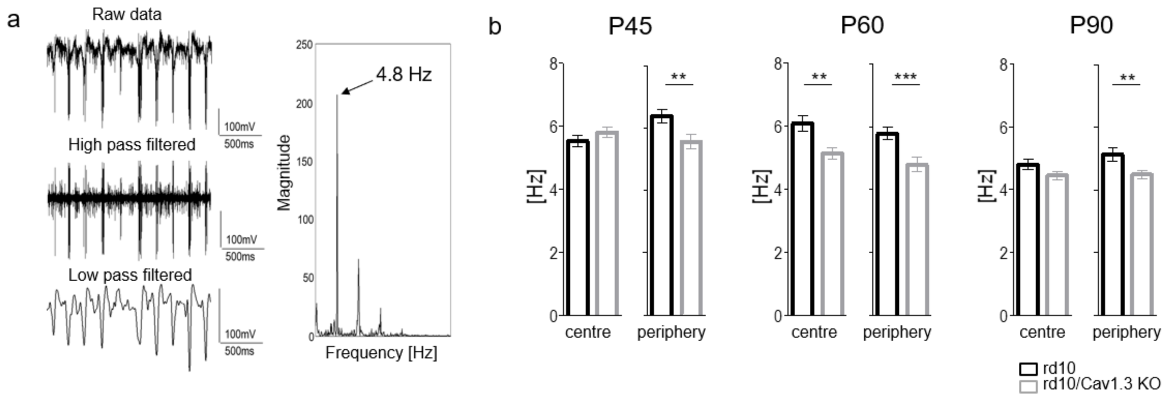

Rhythmic activity was observed in MEA recordings of rd10 and rd10/Cav1.3 KO retina**s. (a)** Representative example of a raw electrical signal recorded from rd10 retina (top), high pass filtered at (300 Hz cut off frequency, middle) and when traces were low pass filtered to obtain the oscillatory activity (50 Hz cut-off frequency, bottom). In the right, the power spectral density obtained by a fast Fourier transformation **(b)** Comparison of spontaneous activity, defined as the highest peak in the Fourier transformation, of rd10 and rd10/Cav1.3-KO retinas recorded either from the centre or the periphery at P45, P60 and P90. Data are presented as means ± SEM. Statistics: Mann Whitney U-test, ***, $p < 0.0001$, **, $p < 0.001$.
